# Predictive, preventive, personalised and participatory periodontology: ‘the 5Ps age’ has already started

**DOI:** 10.1186/1878-5085-4-16

**Published:** 2013-06-14

**Authors:** Carlo Cafiero, Sergio Matarasso

**Affiliations:** 1 University of Naples “FEDERICO II”, Naples 80131, Italy

**Keywords:** Predictive periodontology, Preventive periodontology, Personalised periodontology, Participatory periodontology, Lab-on-a-chip, Gas chromatographs, Cone beam computed tomography, Host-derived diagnostic markers, Saliva, Gingival crevicular fluid

## Abstract

An impressive progress in dentistry has been recorded in the last decades. In order to reconsider guidelines in dentistry, it is required to introduce new concepts of personalised patient treatments: the wave of predictive, preventive and personalised medicine is rapidly incoming in dentistry. Worldwide dentists have to make a big cultural effort in changing the actual ‘reactive’ therapeutic point of view, belonging to the last century, into a futuristic ‘predictive’ one. The first cause of tooth loss in industrialised world is periodontitis, a Gram-negative anaerobic infection whose pathogenesis is genetically determined and characterised by complex immune reactions. Chairside diagnostic tests based on saliva, gingival crevicular fluid and cell sampling are going to be routinely used by periodontists for a new approach to the diagnosis, monitoring, prognosis and management of periodontal patients. The futuristic ‘5Ps’ (predictive, preventive, personalised and participatory periodontology) focuses on early integrated diagnosis (genetic, microbiology, host-derived biomarker detection) and on the active role of the patient in which networked patients will shift from being mere passengers to responsible drivers of their health. In this paper, we intend to propose five diagnostic levels (high-tech diagnostic tools, genetic susceptibility, bacterial infection, host response factors and tissue breakdown-derived products) to be evaluated with the intention to obtain a clear picture of the vulnerability of a single individual to periodontitis in order to organise patient stratification in different categories of risk. Lab-on-a-chip (LOC) technology may soon become an important part of efforts to improve worldwide periodontal health in developed nations as well as in the underserved communities, resource-poor areas and poor countries. The use of LOC devices for periodontal inspection will allow patients to be screened for periodontal diseases in settings other than the periodontist practice, such as at general practitioners, general dentists or dental hygienists. Personalised therapy tailored with respect to the particular medical reality of the specific stratified patient will be the ultimate target to be realised by the 5Ps approach. A long distance has to be covered to reach the above targets, but the pathway has already been clearly outlined.

## Review

### Introduction

**Figure 1:**
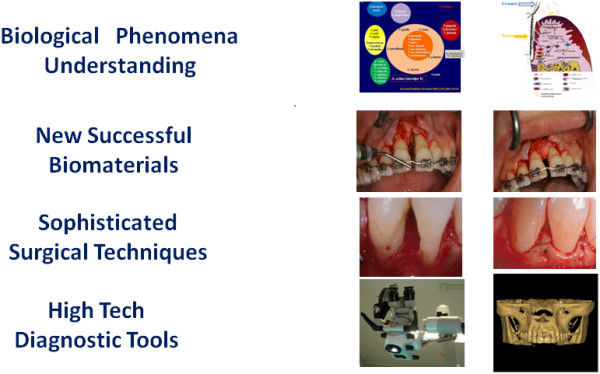
Advances in biological and technical research are clearly outlining the pathway for the future of dentistry.

**Figure 2:**
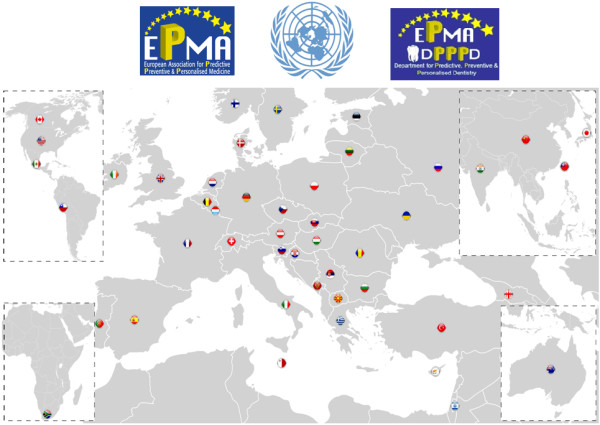
**EPMA is present in 44 countries worldwide.** The EPMA Department for Predictive, Preventive and Personalised Dentistry is going to make a big cultural effort in changing worldwide dentists' therapeutic point of view from a reactive to a predictive one. (Adapted from [1]).

Not too many years ago, the most frequent therapy in dentistry was tooth extraction: the teeth were pulled out and rapidly substituted by the application of a fixed or mobile prosthetic appliance. At that time, due to the weakness of the background in dental researches, just a small number of dentists were able to perform dental therapies on a specialised level. In the majority of cases, advanced therapies were generally considered not more than pioneeristic attempts. An impressive progress in dentistry has been recorded in the last decades. Synergic efforts (understanding of biological phenomena, new biomaterials, sophisticated surgical techniques, high tech in diagnostic tools, etc.) have carried dentistry away from the middle-aged situation described above (Figure 1), but this is not enough. In order to reconsider guidelines in dentistry, it is required to introduce new concepts of personalised patient treatments. On account of this, the current paper follows the recommendations of the recently published ‘White Paper’ of the European Association for Predictive, Preventive and Personalised Medicine (EPMA) [1,2]. The wave of predictive, preventive and personalised medicine is quickly incoming in dentistry. With regard to this, the mission of a specialised EPMA dental section will be to aid worldwide dentists make a big cultural effort in changing the actual ‘reactive’ therapeutic point of view, belonging to the last century, into a futuristic ‘predictive’ one (Figure 2). Enhancement in dental knowledge revealed genetic, microbiological and immunological mechanisms at the base of the most common dental diseases.

**Figure 3:**
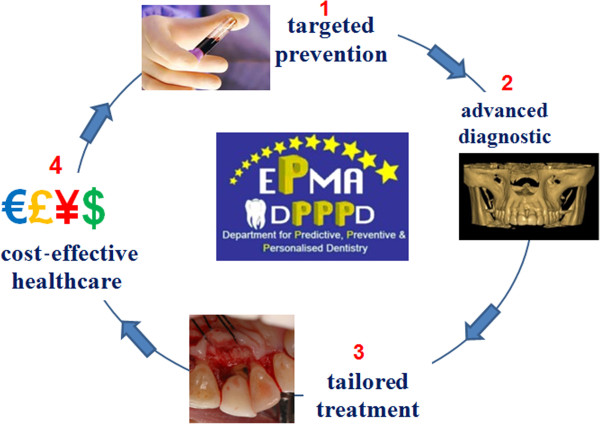
**Since the European population is becoming progressively older, the prevention of periodontitis is of capital importance.** Well-organised screenings have to be performed in order to organise targeted prevention and cost-effective healthcare.

The first cause of tooth loss in industrialised world is periodontitis that strikes prevalently people older than 40 years of age. In consequence of this, the prevention of periodontitis is of capital importance to general and oral health since the European population is becoming progressively older (Figure 3). Periodontitis is a Gram-negative anaerobic infection whose pathogenesis is genetically determined and characterised by complex immune reactions to bacterial burden. It constitutes a very interesting model of chronic oral pathology, characterised by activity phases, related to many branches of medical researches such as genetic, microbiology and immunology. On account of this, chairside diagnostic tests based on saliva, gingival crevicular fluid and cell sampling are going to be routinely used by periodontists for a novel approach to the diagnosis, monitoring, prognosis and management of periodontal patients. As will be discussed later, genetic tests as well as the use of microbial analysis and the detection of biomarkers derived from host response will contribute to improve periodontal health. Predictive, preventive, personalised and participatory periodontology, the ‘5Ps’, represents with no doubt the future of the profession of periodontology. A predictive approach due to the use of high-tech diagnostic tools will give us the possibility to detect patients at risk and to effect early diagnosis of periodontitis when it is easier to treat successfully. It will be organised as a personalised prevention, based upon the genetic and microbiological status of a single patient as well as personalised therapy tailored with respect to the particular medical reality of the specific patient. Finally, the active role of the patient will be emphasised through the introduction of participatory periodontology, a concept in which networked patients will shift from being mere passengers to responsible drivers of their health.

The aim of this paper is to synthetically report actual information on the genetics, microbiology and immunology of periodontal disease related to biomarkers that can aid to have early diagnosis. Further biomarkers, coming out in the early destruction of periodontal tissues, will be equally reported and discussed. The present article is directed not only to dental operators (such as general dentists, periodontists, dental hygienists) but also to medical doctors in order to enlarge the discussion group and to share our experiences and ideas with as much colleagues as possible. Considering the large number of professionals we intend to approach with the present paper, it seems clear that we have to discuss some basic topics about periodontal disease before introducing the specific periodontal biomarkers field.

### Periodontal unit as a multi-functional complex

The periodontium is defined as an anatomic and functional complex which constitutes the supporting tissue of the teeth. Each of the periodontal components has its very specialised function. Periodontal tissues are distinct in the (1) gingiva and (2) deep periodontium (periodontal ligament, cementum and alveolar bone).

#### Gingiva

**Figure 4:**
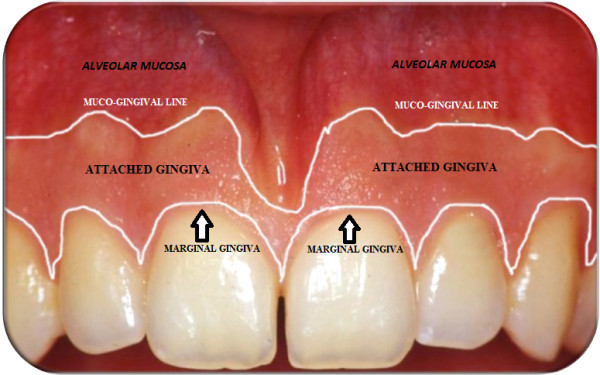
Marginal (free) gingiva, attached gingiva and alveolar mucosa.

The gingiva is a coral pink tissue consisting of an epithelial layer and an underlying connective tissue. It is differentiated into marginal (free) gingiva and attached gingiva. The free gingiva extends at the vestibular and lingual/palatal aspects of the teeth and in the interdental space which constitutes the peak of interdental papillae. On the vestibular and lingual sides, the free gingiva extends from the gingival margin to the free gingival groove which marks the edge with the attached gingiva. The attached gingiva extends in the apical direction to the mucogingival junction where it becomes continuous with the alveolar mucosa [3] (Figure 4).

#### Deep periodontium

**Figure 5:**
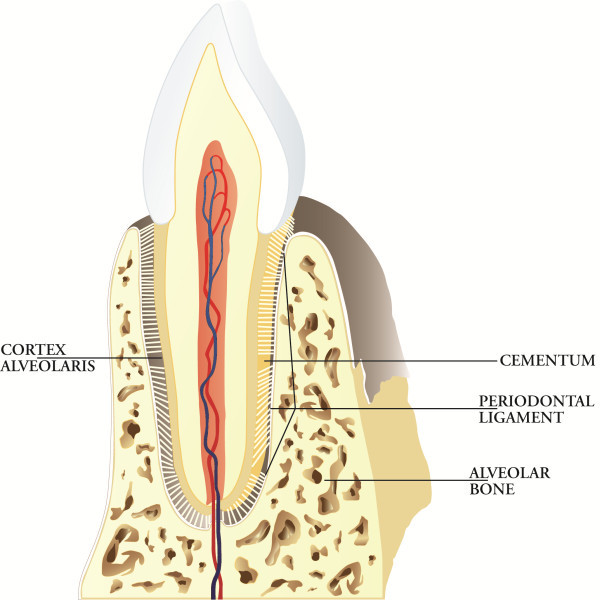
**Deep periodontium.** The main function of the deep periodontium is to support the teeth in their sockets and to act as a sensory receptor necessary for the proper positioning of the jaws and the proper pressure to be exercised during mastication.

The deep periodontium is composed of the periodontal ligament, cementum and alveolar bone (Figure 5).

##### Periodontal ligament

The periodontal ligament (alveolo-dental ligament) is a specialised connective tissue situated between the cementum covering the root of the tooth and the bone forming the socket wall. The extremities of collagen fibre bundles are embedded in the cementum (Sharpey's fibres) on one side and in the alveolar bone on the other side. It ranges in width from 0.15 to 0.38 mm.

##### Cementum

The cementum is the hard, avascular connective tissue covering the roots of the teeth that serves primarily to attach the principal periodontal ligament fibres. There are two principal varieties of the cementum classified on the basis of the presence or absence of cells: acellular extrinsic fibre cementum (primary cementum or acellular cementum) and cellular intrinsic fibre cementum (secondary cementum or cellular cementum). The acellular extrinsic fibre cementum extends from the cervical half to two thirds of the root. The high number of Sharpey's fibres inserting in it shows its fundamental function in tooth attachment. The cellular intrinsic fibre cementum is distributed along the apical third or half of the root and in furcation areas. It represents a reparative tissue.

##### Alveolar bone

The mineralised bone is made up of lamellae (lamellar bone). It includes two types of bone tissue, the bone of the alveolar process and the alveolar bone lining the socket referred to as the alveolar bone proper or ‘bundle bone’ that consists of intrinsic fibre bundles running parallel to the socket. Embedded within this bundle bone and perpendicular to its surface are Sharpey's fibres. The alveolar bone is a clear example of a structure-function relationship because it increases in conjunction with the development of the teeth and it is partially lost in the absence of a tooth. In conclusion, the principal function of deep periodontal tissues is to support the teeth in their sockets. In addition, periodontal tissues act as a sensory receptor necessary for the proper positioning of the jaws and the proper pressure to be exercised during mastication. The peripheral feedback coming from the periodontal ligament gives signals to the muscles, ear and temporomandibular joints about the quality of the food present under the teeth and, as a consequence, the information for the fine-tuning of masticatory pressure.

### Periodontal diseases: the real infections of the oral cavity

Periodontal diseases are a cluster of inflammatory bacterial plaque-induced pathologies. At the end of the 1990s, a classification system for periodontal diseases was proposed, and it is currently used worldwide (see below) [4]:

1. Gingivitis

2. Chronic periodontitis

3. Aggressive periodontitis

4. Periodontitis as a manifestation of systemic disease

5. Necrotising ulcerative gingivitis/periodontitis

6. Abscesses of the periodontium

7. Combined periodontic-endodontic lesions

The most common periodontal diseases are gingivitis and periodontitis whose primary characteristics are synthetically reported below.

#### Gingivitis

**Figure 6:**
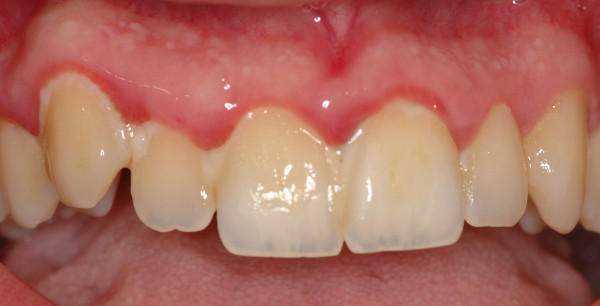
**Upper jaw acute gingivitis in a non-smoker 26-year-old male patient.** Abundant plaque deposit is visible on the surfaces of the teeth.

**Figure 7:**
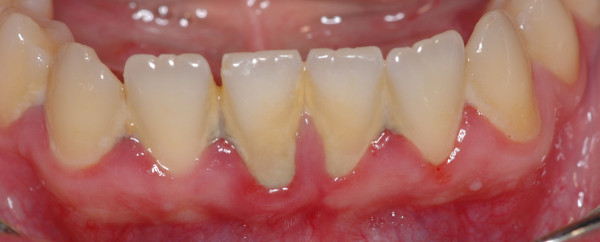
**Lower jaw of the same patient.** Calculus covers the entire surfaces of the teeth.

**Figure 8:**
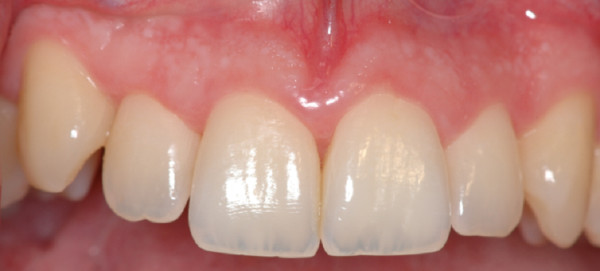
**Upper jaw.** Complete resolution of acute gingivitis and *restitutio ad integrum*.

**Figure 9:**
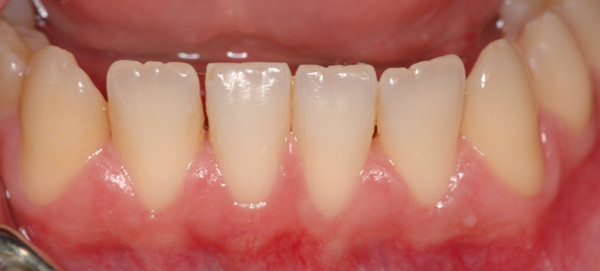
**Lower jaw.** The patient's compliance is of capital importance for a long-term result.

Gingivitis is an inflammation of the periodontal marginal tissue (gingiva) in response to bacterial biofilms (bacterial plaque) adherent to tooth surfaces [5]. It is characterised by redness, bleeding, volume augmentation and diffuse pain (Figures 6, 7, 8, and 9). Gingivitis is a non-destructive periodontal disease in which no deep connective tissue destruction or bone resorption is detectable. In the presence of periodontal treatment, a complete resolution of the disease and *restitutio ad integrum* is to be expected.

#### Periodontitis

**Figure 10:**
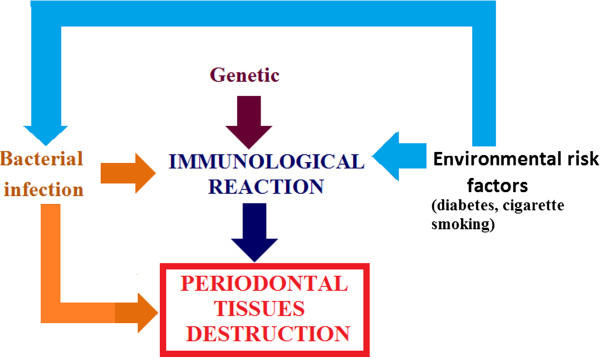
**Periodontitis is a result of a complex bacteria-host response in genetically oriented patients.** Co-factors such as diabetes and cigarette smoking are strongly associated with the aggravation of periodontitis.

Periodontitis is a destructive pathology caused by Gram-negative facultative anaerobes affecting periodontal tissues (gingiva, cementum, periodontal ligament, alveolar bone). It causes periodontal breakdown (connective attachment loss, bone resorption and formation of periodontal pockets) as result of a complex bacteria-host response in genetically oriented patients (Figure 10). Periodontitis is characterised by a cyclic progression in which a recurrent active phase (periodontal breakdown) is followed by a quiescence phase. The natural history of the disease determines progressive periodontal destruction, tooth mobility and migration. This situation can lead to tooth loss and sometimes can render the patient edentulous. Therapy generally stops its progression and in some cases (regenerative surgery) can lead to *restitutio ad integrum*. Periodontitis is classified into chronic and aggressive forms.

##### Chronic periodontitis

**Figure 11:**
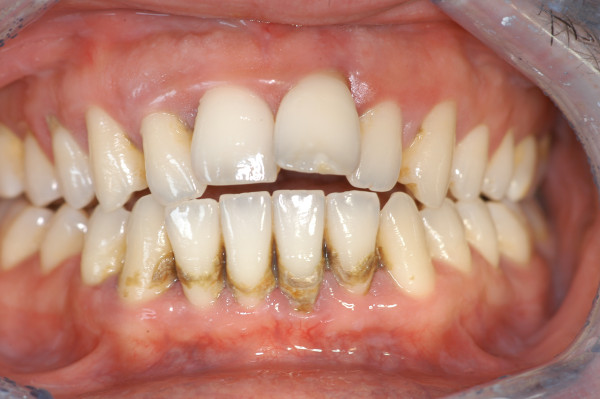
**Generalised chronic periodontitis.** The amount of periodontal tissue destruction commensurate with sub-gingival calculus and plaque amounts, diffuse pathological probing depth, mobility and migration are the main characteristics of this pathology.

**Figure 12:**
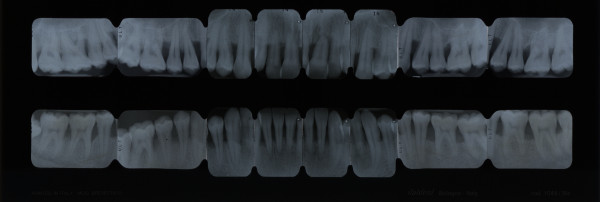
Full-mouth series periapical X-rays show diffuse horizontal bone loss.

Chronic periodontitis (CP) affects up of 50% of the global population, especially older patients, but may occur in children too. In most cases, the rate of progression of chronic periodontitis is slow, and the amount of periodontal tissue destruction is generally commensurate with sub-gingival calculus and plaque amounts. CP is classified as *localised* when <30% of sites are affected and *generalised* when this level is exceeded (Figures 11 and 12).

##### Aggressive periodontitis

Aggressive periodontitis (AP) is less common than the chronic form. In the primary dentition of 5–11-year-olds, the frequency ranges from 0.9% to 1.5% of subjects [6-8], and in the permanent dentition of 12–20-year-olds, the frequency ranges from 0.1% to 0.2% in Caucasian populations. AP generally affects younger patients causing rapid loss of attachment and bone destruction. The severity of periodontal tissue destruction is conflicting with the scarce amounts of microbial deposits. The reason of this destruction is the presence of elevated proportions of aggressive Gram-negative bacteria (*Aggregatibacter actinomycetemcomitans* and *Porphyromonas gingivalis*), the phagocyte abnormalities and the hyperresponsive macrophage phenotype (elevated secretion of prostaglandin E2 (PGE2) and interleukin-1 (IL-1)) in response to bacterial lipopolysaccharides (LPSs). Aggressive periodontitis has been sub-classified into localised and generalised forms [9,10].

##### Localised aggressive periodontitis

Localised aggressive periodontitis (LAP) presents a circumpubertal onset. The first molar/incisor presents with interproximal attachment loss on at least two permanent teeth, one of which is the first molar, and involving no more than two teeth other than the first molars and incisors. Serum antibody response to infecting agents was detected in high quantity.

##### Generalised aggressive periodontitis

**Figure 13:**
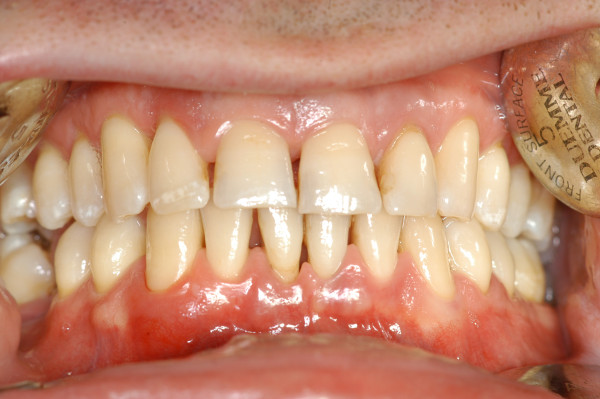
**Generalised aggressive periodontitis.** The scarce amount of microbial deposits is conflicting with the severity of periodontal tissue destruction as shown in Figure 14.

**Figure 14:**
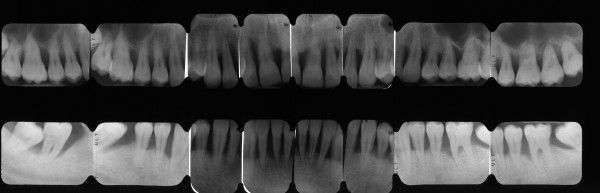
**Full-mouth series periapical X-rays.** Advanced bone destruction is evident.

Generalised aggressive periodontitis (GAP), formerly named generalised early-onset periodontitis, usually affects patients aged under 30. GAP is characterised by the presence of generalised interproximal attachment loss affecting at least three permanent teeth other than the first molars and incisors [11] (Figures 13 and 14).

### The genetic bases of periodontitis

The observation that inheritance was an important component in the development of periodontal diseases was proposed as early as 1935 [12]. Investigations focused on genetic risk factors are currently characterising periodontal research in the genetic field.

#### Mutations and polymorphisms

DNA sequence variations are described as mutations and as polymorphisms. A mutation is defined as a change in a DNA sequence away from normal. In periodontitis, specific mutations have been identified to cause the genetic basis of various syndromic conditions (Table 1) [13]. Genetic mutations are deterministic of simple genetic diseases. However, these genetic diseases are rare and do not characterise the most common forms of periodontitis. In fact, from a genetical point of view, probably the most common forms of periodontitis arise as a result of single-nucleotide polymorphisms (SNPs). The arbitrary cut-off point between a mutation and a polymorphism is 1%. That is, to be classed as a polymorphism, the least common allele must have a frequency of 1% or more in the population. When the frequency is lower than this, the allele is regarded as a mutation. Numerous studies have investigated SNPs in both the chronic and aggressive forms of periodontitis. The distribution of SNPs varied between ethnic groups. Ethnic difference(s) in genes encoding the interleukins IL-1 and IL-6, Fc receptors (Fc RIIa, Fc RIIIa and Fc RIIIb), tumour necrosis factor alpha (TNF-α), vitamin D receptor, CD14 and matrix metalloproteinase-1 (MMP-1) were found. Several studies investigated the genes affecting the expression of interleukin-1, interleukin-6, tumour necrosis factor, interleukin-10, E-selectins, Fc-gamma receptor, CD14, toll-like receptors and periodontal disease. The largest part of the studies shows no correlations between the presence of disease markers and the tested SNP in both the aggressive and chronic forms of periodontitis [14]. It is important to clarify that in complex disease, a given polymorphism is necessary but not sufficient to cause disease. In fact, the interplay of genetic and environmental factors is fundamental in determining the disease phenotype. Hence, a single functional genetic polymorphism is not sufficient to cause disease; consequently, such functional polymorphisms may be found in individuals with no evidence of disease.

**Table 1:** Specific mutations cause the genetic basis of various syndromic conditions in which periodontitis is present

**Disease**	**Biochemical/tissue defect**	**Inheritance**
Papillon-Lefèvre syndrome	Cathepsin C	Autosomal recessive
Chédiak-Higashi syndrome	Lysosomal trafficking regulator gene	Autosomal recessive
Leukocyte adhesion deficiency type 1	Leukocyte chain adhesion molecule CD18	Autosomal recessive
Leukocyte adhesion deficiency type 2	Glucose diphosphatase-fucose transporter-1	Autosomal recessive
Cyclic neutropenia	Neutrophil elastase	Autosomal dominant
Haim-Munk syndrome	Cathepsin C	Autosomal recessive
Ehlers-Danlos syndrome	Collagen	Autosomal dominant

### Bacterial burden: a challenge for periodontal tissues

The average 200-lb (90 kg) human body carries around with it about 6 lb (2.7 kg) of bacteria. Some of them live in the oral cavity forming a huge source of bacteria: to give an idea, in 1 mm^3^ (1 mg) of dental plaque, 10^8^ bacteria are present. The Human Oral Microbiome Database lists 1,200 predominant oral species, with the order of 19,000 phylotypes [15].

Dental plaque is a biofilm containing over 700 individual taxa of aggregated microorganisms embedded within a self-produced matrix of extracellular substance composed of bacterial polymers and salivary and gingival exudate products (Table 2). The heterogeneity of plaque gradually increases and includes large numbers of Gram-negative anaerobic species in gingivitis (approximately 25%) and periodontitis (approximately 75%) as compared to healthy gingiva (approximately 13%) (Table 3).

**Table 2:** Principal constituents of dental plaque

	**Constituents**	
Bacterial aggregate	Gram-positive	
	Gram-negative	
Matrix of extracellular substance	Glucides	Levans
		Dextrans
		Glycogen
		Galactose
		Ribose
		Fucose
	Lipids	Glycolipids
		Phospholipids
		Triglycerides
		Cholesterol
	Proteins	Glycoproteins
		Glycosaminoglycans
		Lipoproteins
	Ions and trace elements	Ca^++^/PO_4_^−−^
		F^−^
		Ag, Mg, Co, Fe, Cu, Pb, Sn

**Table 3:** The most frequent microbial species isolated in healthy gingiva, gingivitis and periodontitis

	**Microbial species**
Healthy gingiva	*Streptococcus oralis*
	*Streptococcus sanguis*
	*Streptococcus mitis*
	*Streptococcus gordonii*
	*Streptococcus mutans*
	*Streptococcus anginosus*
	*Streptococcus intermedius*
	*Gemella morbillorum*
	*Rothia dentocariosa*
	*Actinomyces naeslundii*
	*Actinomyces gerencseriae*
	*Actinomyces odontolyticus*
	*Peptostretococcus micros*
	*Eubacterium nodatum*
	*Capnocytophaga ochracea*
	*Capnocytophaga gingivalis*
	*Campylobacter gracilis*
	*Fusobacterium nucleatum* subsp. *polymorphum*
Gingivitis	*Streptococcus oralis*
	*Streptococcus sanguis*
	*Streptococcus mitis*
	*Streptococcus intermedius*
	*Capnocytophaga ochracea*
	*Capnocytophaga gingivalis*
	*Campylobacter gracilis*
	*Prevotella loescheii*
	*Peptostreptococcus micros*
	*Eubacterium nodatum*
	*Actinomyces naeslundii*
	*Actinomyces israelii*
	*Campylobacter concisus*
	*Actinomyces odontolycus*
	*Fusobacterium nucleatum* subsp. *nucleatum*
	*Eubacterium brachy*
	*Eikenella corrodens*
	*Aggregatibacter actinomycetemcomitans* serotype a
Periodontitis	*Porphyromonas gingivalis*
	*Aggregatibacter actinomycetemcomitans* serotype b
	*Bacteroides forsythus*
	*PRO spirochete*
	*Treponema denticola*
	*Prevotella intermedia*
	*Prevotella nigrescens*
	*Campylobacter rectus*
	*Peptostreptococcus micros*
	*Fusobacterium nucleatum* subsp. *vincentii*
	*Fusobacterium nucleatum* subsp. *nucleatum*
	*Selenomonas noxia*
	*Selenomonas flueggeii*
	*Enteric* species
	*Fusobacterium alocis*
	*Lactobacillus uli*
	*Veillonella parvula*

Further and more recent studies have demonstrated that there are specific associations among bacterial species within dental plaque.

**Figure 15:**
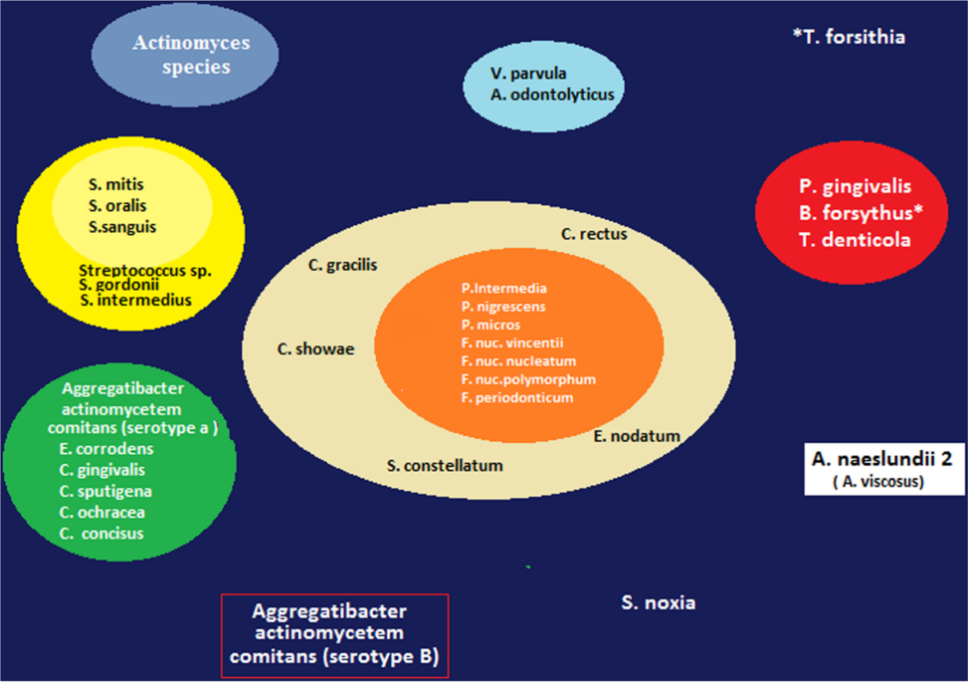
**Specific associations among bacterial species within dental plaque characterise five closely associated clusters.** (Adapted from [16]).

Five closely associated clusters have been reported (Figure 15):

1. The green cluster (*Campylobacter concisus*, *Eikenella corrodens*, *Actinobacillus actinomycetemcomitans* serotype a).

2. The yellow cluster made up of a group of streptococci (*Streptococcus mitis*, *Streptococcus sanguis*, *Streptococcus oralis*).

3. The purple cluster (*Actinomyces odontolyticus*, *Veillonella parvula*).

4. The red cluster (*P. gingivalis*, *Tanerella forsythia*, *Treponema denticola*).

5. The orange cluster (*Fusobacterium nucleatum* subspecies, *Prevotella intermedia*, *Prevotella nigrescens*, *Peptostreptococcus micros*, *Campylobacter rectus*, *Campylobacter showae*, *Campylobacter gracilis*, *Eubacterium nodatum*, *Streptococcus constellatus*, *Fusobacterium periodonticum*).

Finally, *Actinotmyces naeslundii* genospecies 2 (*Actinomyces viscosus*), *Selenomonas noxia* and *A. actinomycetemcomitans* serotype b did not cluster with other species [16].

**Figure 16:**
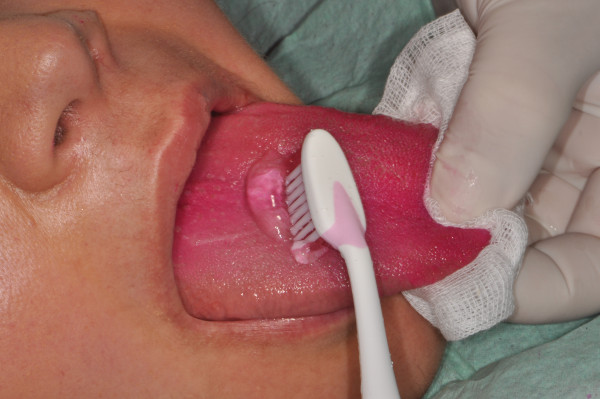
**Tongue dorsum brushing with 0.5%chlorhexidine gel.** The red colour of the tongue is due to the use of erythrosine pads that have the capability to reveal the presence of bacterial plaque on the teeth and soft tissue. Chlorhexidine (0.12%) puffs on the tonsils and chlorhexidine (0.12%) mouth rinse are further procedures necessary to eradicate periodontal pathogens from the whole mouth.

Periodontopathogens also colonise non-dental surfaces such as the tongue dorsum, oral mucosa and tonsils, and for this reason, periodontitis therapeutic measures have to eliminate periodontal pathogens in the whole mouth (Figure 16) [17].

An epidemiologic study found out that close members of the same family were infected via saliva with *A. actinomycetemcomitans* strains of the same biotype and serotype [18]. For this reason, prevention measures against periodontal pathogens must include the entire family members in order to prevent cross-infection [19].

Even if there are no sufficient microbiological evidences that could help us distinguish the different forms of periodontitis, it is clear that:

1. The chronic and aggressive forms of periodontitis are not monoinfections.

2. Some microbiota are more important than others as aetiological agents of periodontitis.

### Periodontal tissues as a ‘battlefield’ in the struggle against oral bacteria

The host-microbial balance constitutes the situation in clinically healthy periodontal tissue. Plaque accumulation and immunitary response can create an imbalance of the host-parasite relationship occurring in destructive periodontal lesion. In fact, the fight among bacteria and immunocompetent cells can devastate the battlefield, that is, the periodontium.

**Figure 17:**
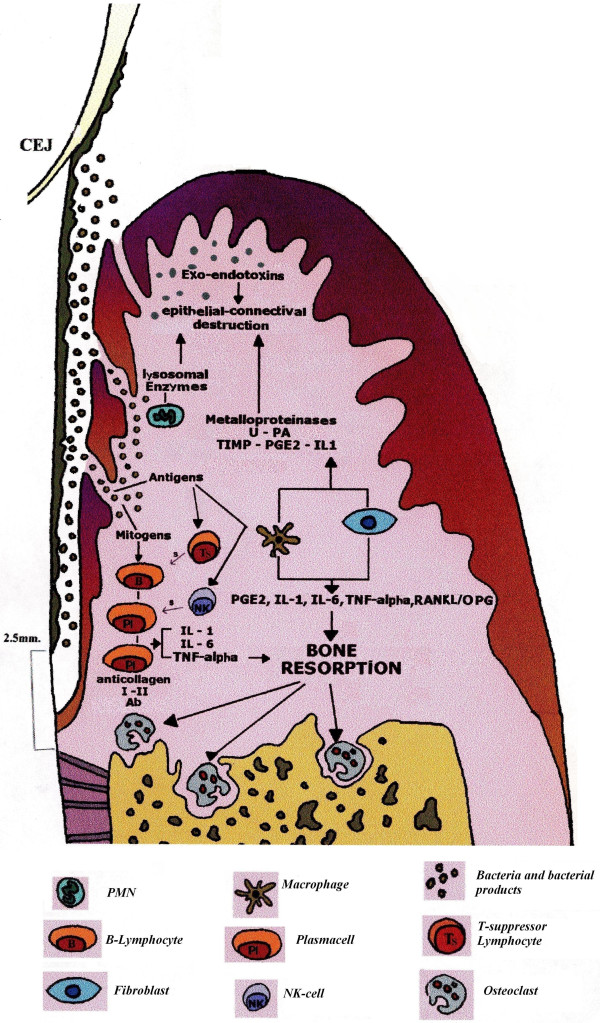
**The mortal fight among bacteria and immunocompetent cells.** It can devastate the ‘periodontal battlefield’ since defensive immunitary reaction could paradoxically contribute to the tissue destruction. Activated polymorphonuclear leukocytes, indeed, can cause tissue damage as a result of a variety of enzymes and oxygen metabolites that are released from their granules during the battle against microbes. Bacterial LPSs activate macrophages, lymphocytes and fibroblasts which secrete lymphokines activating MMPs. Metalloproteinases are enzymes that degrade the connective extracellular matrix and can be detected in gingival crevicular fluid. Finally, many substances (PGE2, IL-1, IL-6, TNF-α) secreted by Mø, fibroblasts, plasma cells and T lymphocytes are primarily involved in osteoclastic activation via the RANKL-OPG expression system.

For simplification, we arbitrarily divided the immunitary response in periodontal tissue into three different compartments (epithelium, connective, bone) in which three main cells (polymorphonuclear neutrophils (PMNs), macrophages, osteoclasts) are representative of three different topical moments (Figure 17).

#### Epithelial compartment: PMN activation

PMN leukocytes represent the first line of defence forming a protective wall against microorganisms. Activated polymorphonuclear leukocytes can cause tissue damage as a result of their accumulation in epithelial tissues. Further damages can be caused by a variety of enzymes and oxygen metabolites that are released from their granules during the battle against microbes [20,21]. Oxygen metabolites such as hydrogen peroxide (H_2_O_2_) and reactive oxygen radicals (OH^−^) that are released into the phagosome defensive immunitary reaction could paradoxically contribute to the tissue destruction. As a consequence, the junctional epithelium becomes filled with ulcers and allows the passage of bacteria and their products in the underneath connective tissue.

#### Connective compartment: macrophage activation

In the subsequent line of defence, macrophages (Mø) play a decisive role to restrict bacterial spreading in the connective tissue. Macrophages are an important source of lysosomal enzymes, cytokines and inflammatory mediators such as IL-1, TNF-α, PGE2 and transforming growth factor beta (TGF-β).

IL-1 is the principal inflammatory mediator released by LPS-activated macrophages, lymphocytes and fibroblasts. IL-1 stimulates Mø and fibroblasts to secrete PGE2; moreover, it causes osteoclastic differentiation and activation [22].

TNF-α, principally secreted by LPS-stimulated macrophages and lymphocytes, causes osteoclastic differentiation and activation [23].

PGE2 causes vasodilatation, vasopermeability and resorption of the alveolar bone. IL-1, TNF-α and PGE2 stimulate fibroblasts and Mø to release MMPs, urokinase plasminogen activator (u-PA), tissue inhibitor of metalloproteinases, PGE2, TGF-β and interleukin-1 receptor antagonist. As described below, disease severity appears linked to the existent equilibrium among different involved molecules. The u-PA causes plasminogen transformation in plasmin which activates MMPs, enzymes degrading the connective extracellular matrix. They can be detected in gingival crevicular fluid, particularly during the activity phase [24].

#### Bone compartment: osteoclast activation

Inflammation progresses in the apical direction involving the bone tissue. It is important here to highlight that bacterial plaque never gets in direct contact with the bone tissue and that ‘running away’ from the bacterial aggregate is always at least 2 mm in distance from it. Many substances (PGE2, IL-1, IL-6, TNF-α) secreted by Mø, fibroblasts, plasma cells and T lymphocytes are primarily involved in osteoclastic activation.

**Figure 18:**
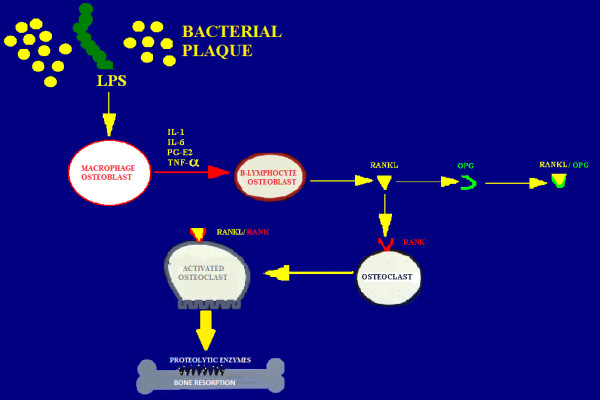
**The RANKL-OPG expression system.** Under physiological condition, RANKL produced by osteoblasts binds to RANK on the surface of osteoclast precursors. OPG is produced by fibroblasts constituting a false target for RANKL. The balanced regulation of the RANKL-OPG expression system can determine health from disease.

The receptor activator of NF-kB ligand (RANKL) is a recently described member of the tumour necrosis factor superfamily promoting osteoclastic differentiation from haemopoietic precursors and the inhibition of osteoclast apoptosis. Under physiological condition, RANKL produced by osteoblasts binds to RANK on the surface of osteoclast precursors. RANKL is up-regulated by osteotropic factors such as PTH and IL-11. Osteoprotegerin (OPG), a member of the TNF receptor superfamily, is produced by fibroblasts constituting a false target for RANKL [25,26]. Hence, OPG is an inhibitor of bone resorption. The balanced regulation of the RANKL-OPG expression system can determine health from disease (Figure 18).

### Environmental risk factors

Smoking and diabetes mellitus are the most frequent co-factors strongly associated with the aggravation of periodontitis. Other situations such as obesity, stress and osteoporosis have been identified as co-factors in the progression of periodontitis [27].

#### Diabetes mellitus

**Figure 19:**
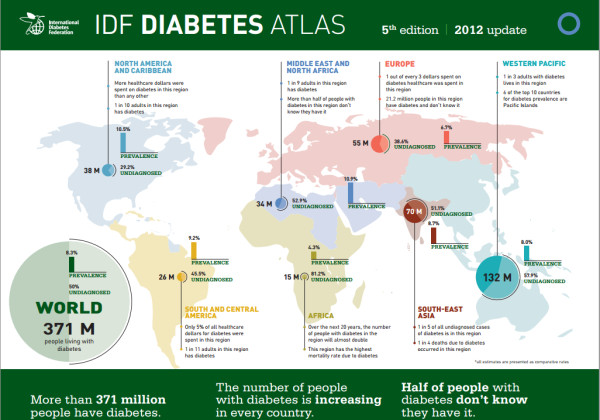
**More than 371 million people have diabetes worldwide and the number is increasing in every country.** It is important to highlight that half of the people with diabetes do not know that they have it, and for this reason, the majority of people who die from diabetes are under the age of 60. Nearly 5 million people died and US$471 billion were spent due to diabetes in 2012. International organisations are going to face a dramatic diabetic emergency in the next years. (Adapted from [28]).

The present percentage of diabetics is very high worldwide, and these numbers are increasing dramatically. It is not exaggerate to claim that we are going to face a dramatic diabetic emergency in the next years (Figure 19).

The contemporary consensus is that diabetic patients are at increased risk of periodontitis [29]. Patients with type 1 (insulin-dependent) and type 2 (non-insulin-dependent) diabetes mellitus have been found to be equally at risk for periodontitis [30]. The severity of periodontitis has been proved to increase with the onset of diabetes at a younger age as well as with poorer metabolic control of diabetes [31]. It has been claimed that periodontitis is the sixth complication of diabetes, together with retinopathy, nephropathy, neuropathy, macrovascular diseases and altered wound healing [32]. Diabetes mellitus is the only systemic disease positively associated with attachment loss with an odds ratio of 2.32 (95% confidence interval (CI) 1.17–4.60) [33]. Some authors presumed a two-way relationship in which periodontal therapy can improve metabolic control in diabetic patients [34]. In these studies, periodontal treatment was associated with a reduction in HbA1c levels, and moreover, inflammatory biomarkers decline with periodontal treatment [35-37]. In contrast, non-significant reduction in HbA1c values was recorded in several studies [38-40]. Very recently, a meta-analysis of nine intervention studies of 485 people with diabetes concluded that periodontal treatment could lead to a significant 0.79% (95% CI 0.19–1.40) reduction in HbA1c levels [41]. A recent Cochrane review on the treatment of periodontal disease for glycaemic control in people with diabetes declared that further controlled studies are necessary to clarify the topic [42]. These conflicting data are difficult to understand in order to clarify the influence of periodontitis in glycaemic control. Hence, supplementary controlled clinical trials appear urgent and necessary to definitely assess if periodontal therapy can improve metabolic control in diabetic patients.

#### Smoking

Over the past decades, a multitude of papers about the relationship between smoking and periodontitis have been published. The contemporary consensus is that:

1. Cigarette smoking is associated with a relative risk, ranging from 2.05 (95% CI 1.47–2.87) for light smokers increasing to 4.75 (95% CI 3.28–6.91) for heavy smokers, of developing periodontitis [33,43].

2. The negative effect of smoking is dose dependent and cumulative [44].

3. The negative effect of smoking is marked in younger individuals [45].

4. Smoking affects the healing potential of periodontal tissues [46].

5. Smoking is associated with the recurrence of periodontitis during periodontal maintenance [47].

#### Obesity, stress and osteoporosis

Other conditions such as obesity, stress and osteoporosis have been involved as co-factors in the progression of periodontitis, even if the association appears weak and still debatable.

##### Obesity

It has been suggested that obesity is a strong risk factor for periodontal tissue destruction [48] since adipose tissue represents much more than a fat accumulation. It produces cytokines and hormones, collectively called adipokines or adipocytokines, which may play a key role in modulating periodontitis [49].

An association between obesity and periodontal disease in humans was reported for the first time by Saito et al. [50]. The authors estimated that the relative risk for periodontitis was 3.4 in persons with a body mass index of 25–29.9 kg/m^2^ and 8.6 in those with a body mass index of >30 kg/m^2^. These results were confirmed by other authors [51,52]. Genco et al. [53] demonstrated that the severity of periodontal attachment loss was modulated by insulin resistance. In addition, it was reported that maintaining a normal weight was associated with a poorer frequency of periodontitis [54,55].

##### Stress

The impact of stress on periodontal diseases has not yet been clarified. Stressful life events have been shown to modulate the endocrine and immune systems. Stressful life events could affect periodontal disease progression through (1) unhealthy behaviours (poor oral hygiene, increased tobacco smoking) and (2) pathophysiological factors (higher glucocorticoid and catecholamine levels) which affect bacterial, immunological, inflammatory and hormonal profiles, leading to an increased susceptibility to periodontal disease [56,57]. Finally, in a systematic review, a positive relationship between stress and chronic periodontitis was confirmed [58].

##### Osteoporosis

Osteoporosis is a metabolic bone disorder characterised by the loss of bone mineral density, principally recorded in postmenopausal women. It has been proposed that osteoporosis could affect the alveolar bone leading to rapid resorption in periodontal women. In one study, 189 postmenopausal women were controlled over a 7-year period. An association between the loss of bone mineral density and increased risk of additional tooth loss was reported. In a review, it has been shown that 7 out of 17 studies reported a positive relationship between osteoporosis and clinical attachment loss. Eleven out of 19 studies found a positive association between osteoporosis and tooth loss [59]. Other studies showed negative or equivocal results [60].

It can therefore be concluded that since many of the studies were uncontrolled and had small sample sizes, the validity of their conclusions needs to be confirmed. Thus, the association between osteoporosis and periodontitis in humans remains weak and still debatable [61].

### Periodontology approaches the future: 5Ps for five diagnostic levels

In addition to the traditional instruments for periodontal diagnosis, in the next future, well-organised population screening protocols utilising chairside diagnostic biomarkers for periodontal disease will be disposable. With reference to this, the last section of the present paper will be focusing on the diagnostic tools currently utilised for periodontal diagnosis (*the present time*) and on the most promising diagnostic tools (i.e. biomarkers and high-tech instrumentations) that are going to enter in clinical periodontology (*the next future*).

#### The present time: a precise picture of a single periodontal patient's existing condition

Diagnostic imaging and periodontal charting provide a complete description of the patient's periodontal condition.

#### Diagnostic imaging: a fundamental step to assess the periodontal conditions of a single patient

**Figure 20:**
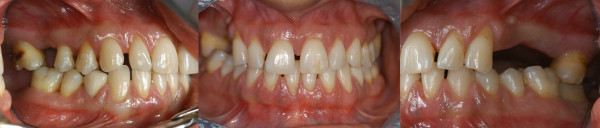
**Full-mouth high-definition digital photographs.** By the use of a high-resolution professional digital camera, the operator takes a series of pictures during the initial visit. Before and after pictures can give periodontists and patients an objective representation of periodontal health improvement. Thus, the camera is a fantastic educational aid to reinforce the compliance of the patients and a diagnostic tool for the periodontist.

**Full-mouth high-definition digital photographs** By the use of a high-resolution professional digital camera, the operator takes a series of five pictures (frontal, right lateral, left lateral, palatal and lingual sides) during the initial visit. Since the camera is used in tandem with a computer screen, we can, in real time, easily show the patient the recorded images to illustrate his/her dental and periodontal health. The camera is not only a diagnostic tool but also a fantastic educational aid in helping us to reinforce the *compliance* of the patients, one of the most important topics in participatory periodontology. Finally, ‘before and after’ pictures can give periodontists and patients an objective representation of periodontal health improvement (Figure 20).

##### Full-mouth series periapical X-rays

**Figure 21:**
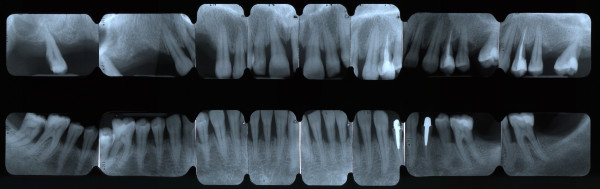
**A full-mouth X-ray series.** It is an important diagnostic support in periodontal patients (14/16 periapical X-rays) since it creates a full view of the patient's teeth and surrounding bone tissue.

An intra-oral X-ray provides a clear picture of the state of the patient's individual tooth from the crown to the tip of its root (Figure 21). Moreover, it provides information on the height and configuration of the interproximal alveolar bone. A full-mouth X-ray series is an important diagnostic support in periodontal patients (14/16 periapical X-rays). Full-mouth series periapical X-rays create a full view of the patient's teeth and surrounding bone tissue which must be combined with a meticulous assessment of periodontal charting in order to make a correct evaluation regarding ‘horizontal’ and ‘angular’ bony defects.

##### Periodontal charting: a complete status of the patient's periodontal health

**Figure 22:**
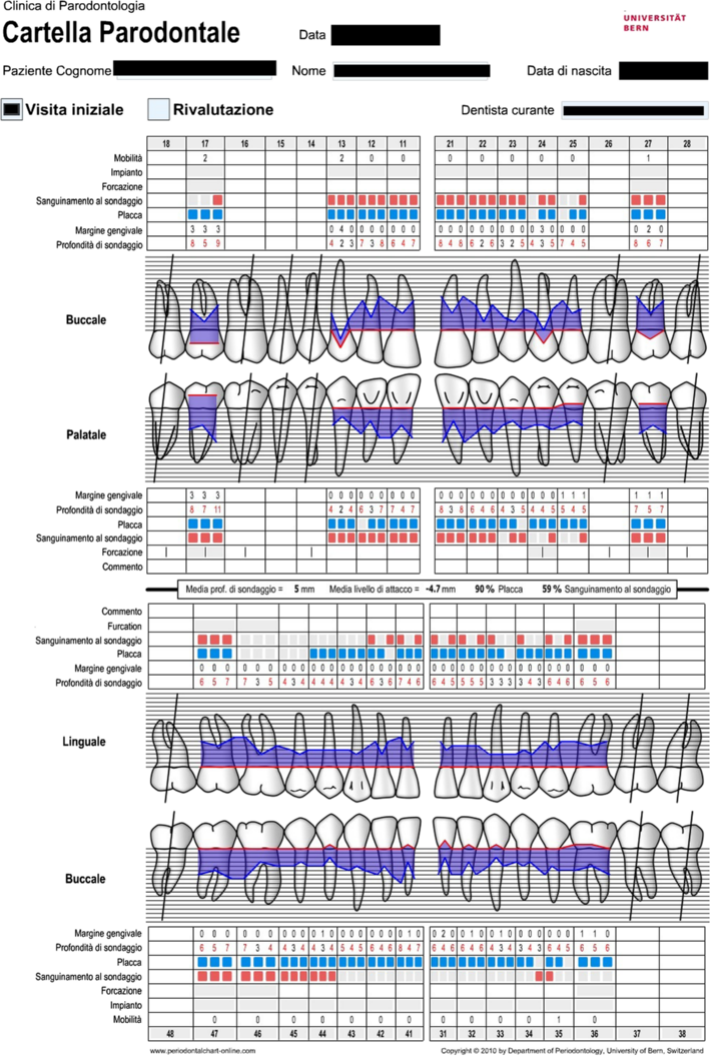
**Periodontal charting provides a complete picture of the periodontal conditions of a single patient.** (Adapted from [62]).

**Figure 23:**
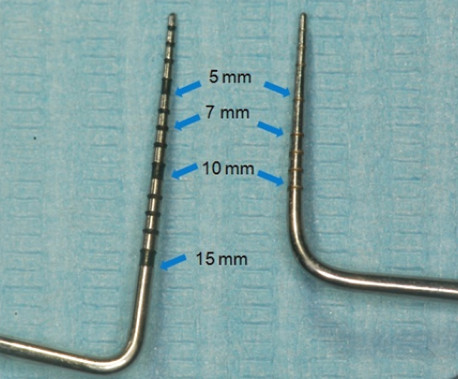
Calibrated periodontal probes are routinely used for periodontal screening.

**Figure 24:**
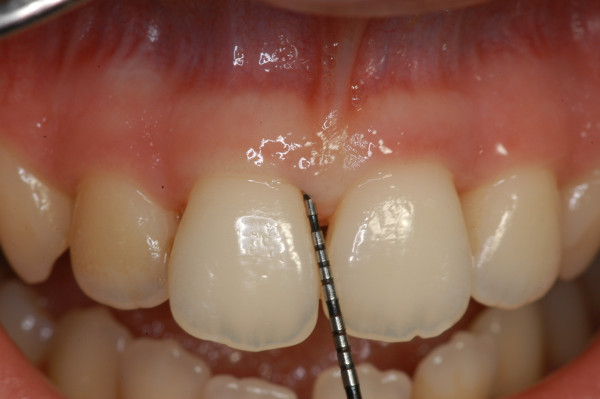
**A periodontal probe.** It is inserted into the sulcus and in a parallel position with respect to the long axis of the tooth. The physiological value of PPD is considered to be ≤3 mm. PPD allows an immediate evaluation of diseased sites.

Periodontal charting (full-mouth plaque score, full-mouth bleeding score, probing depth, clinical attachment level, bleeding on probing, recessions, mobility, migration, halitosis) provides a complete picture of periodontal conditions of a single patient (Figure 22) [3]. Measurements are accomplished with a calibrated periodontal probe (Figure 23) inserted into the sulcus and in a parallel position with respect to the long axis of the tooth (Figure 24).

##### Full-mouth plaque score

The full-mouth plaque score is defined as the percentage of sites where plaque is present divided by the number of sites examined.

##### Full-mouth bleeding score

The full-mouth bleeding score is defined as the percentage of sites bleeding with respect to the number of sites examined.

##### Probing pocket depth

**Figure 25:**
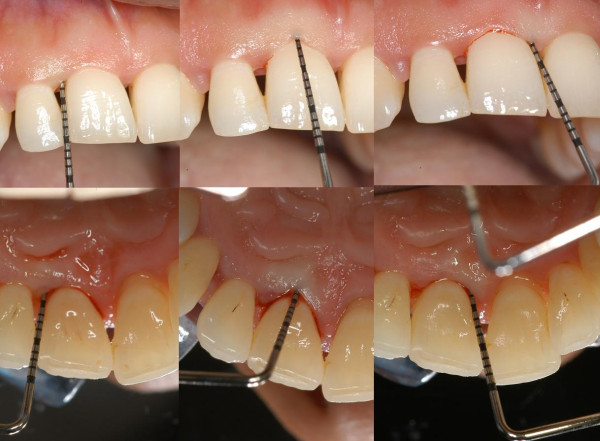
**PPD and CAL measurements.** They are taken for each tooth at (*left* to *right*) the mesio-buccal line angle, the mid-buccal, the distobuccal line angle, the distolingual line angle, the mid-lingual and the mesio-lingual line.

**Figure 26:**
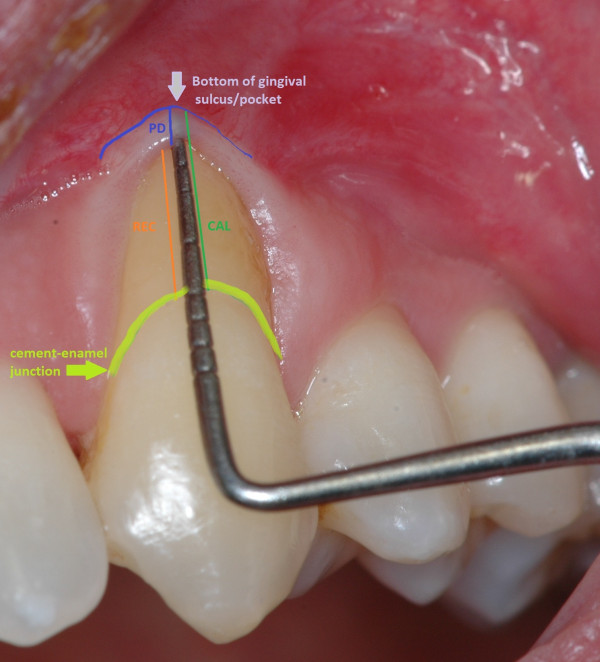
**PPD, CAL and REC measurements.** PPD (*blue line*) is the distance from the gingival margin to the bottom of the gingival sulcus/pocket. CAL (*green line*) is assessed by means of a graduated probe and expressed as the distance in millimetres from the CEJ to the bottom of the periodontal pocket. REC (*orange line*) is defined as the apical migration of the gingival margin. It is measured from the cement-enamel junction (*curved yellow green line*) to the gingival margin.

Probing pocket depth (PPD) is the distance from the gingival margin to the bottom of the gingival sulcus/pocket. It is measured by means of a graduated periodontal probe with a standardised tip diameter of 0.5 mm. Measurement is taken for each tooth at the mesio-buccal line angle, the mid-buccal, the distobuccal line angle, the distolingual line angle, the mid-lingual and the mesio-lingual line (six sites for each tooth) (Figures 25 and 26). The physiological value of PPD is considered to be ≤3 mm. PPD allows an immediate evaluation of diseased sites.

##### Clinical attachment level

Clinical attachment level (CAL), formerly called probing attachment level, is assessed by means of a graduated probe and expressed as the distance in millimetres from the cement-enamel junction (CEJ) to the bottom of the periodontal pocket (Figures 25 and 26). The severity of the attachment loss may be considered mild (CAL = 1–2 mm), moderate (CAL = 3–4 mm) or severe (CAL ≥ 5 mm).

##### Recessions

Recession (REC) is defined as the apical migration of the gingival margin. In most cases, it is due to gingival inflammation or incorrect (traumatic) tooth brushing. It is measured from the cement-enamel junction to the gingival margin by the use of a periodontal probe (Figure 26).

##### Bleeding on probing

**Figure 27:**
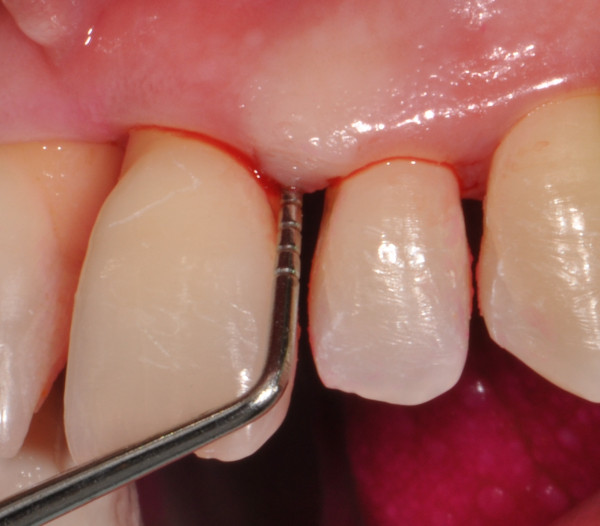
Blood coming out from the bottom of the pocket can be recorded during probing (BoP+).

A periodontal probe is inserted at the ‘bottom’ of the gingival sulcus or periodontal pocket. Blood coming out from the bottom of the pocket can be recorded during probing (Figure 27). Bleeding on probing (BoP) is currently the unique predictive test routinely used for monitoring disease progression or periodontal stability (discussed in the next sections).

##### Mobility and migration

Unphysiological mobility and migration are generally due to the reduction of periodontal support caused by bone resorption in consequence of periodontitis. Physiological forces (tongue, lips, occlusion, etc.) can cause the movement and migration of the tooth with reduced periodontium.

##### Halitosis

Halitosis is defined as the presence of unpleasant breath odour*.* Gram-negative bacteria are the primary pathogens responsible for oral malodour production. Other causes of halitosis are uncontrolled diabetes, gastrointestinal diseases, renal failure and diseases affecting the upper/lower respiratory tract.

**Figure 28:**
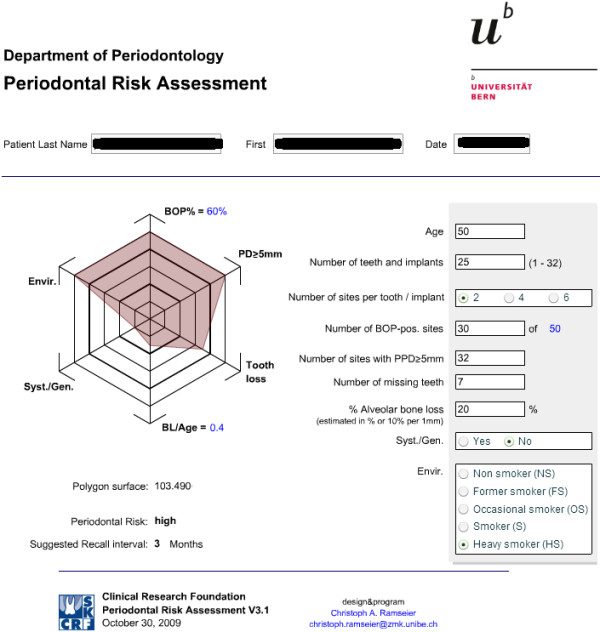
**Spider's web.** It consists of an assessment of the level of infection of a single patient contemplated and evaluated together. In the present case, a heavy-smoker 50-year-old patient presents a high periodontal risk (30 BOP + sites, 32 sites with PPD ≥ 5 mm). (Adapted from [67]).

**Figure 29:**
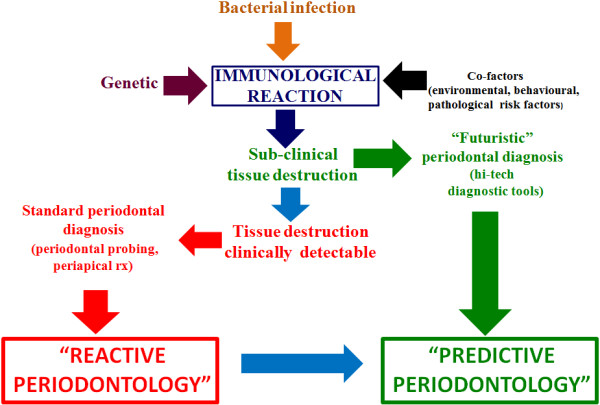
**The aim of predictive, preventive, personalised, participatory periodontology.** The aim is to transform the actual reactive therapeutic point of view, in which tissues destruction is clinically detectable, into a futuristic predictive one in which the disease is early intercepted when it is already in a sub-clinical phase.

Unfortunately, periodontists can get only few predictive information about the progression and none about the rise of the disease from the tools described above. BoP is currently the unique predictive test used by periodontists for routinely monitoring disease progression or periodontal stability. BoP repeatedly positive (BoP+) is a predictor of future loss of attachment (activity phase) in 30% of cases (positive predictive value), meanwhile BoP repeatedly negative (BoP−) is a predictor of periodontal health in 98% of cases (negative predictive value) [63-65]. In addition to that, a functional diagram to evaluate the patient's risk for recurrence of periodontitis (‘spider's web’) has been proposed (Figure 28). It consists of an assessment of the level of infection (full-mouth bleeding score), the prevalence of residual periodontal pockets, tooth loss, an estimation of the loss of periodontal support in relation to the patient's age, an evaluation of the systemic conditions of the patient and finally an evaluation of environmental and behavioural factors such as smoking. All these factors should be contemplated and evaluated together [66]. Bearing in mind what has been discussed above, it appears clear that, at present, a periodontal defence strategy is almost totally reactive: periodontists take action generally when periodontitis has already begun in periodontium destruction. In order to face mild or advanced periodontal lesions, periodontists are currently able to put in place sophisticated periodontal therapeutic strategies, but this does not seem enough. Recently, researches are gradually giving us the instruments to switch the therapeutic point of view from the current reactive to a more advanced predictive model (Figure 29).

#### The next future: hi-tech diagnostic tools and specific biomarkers to detect early periodontal damage

Knowledge in dentistry is estimated to double every 4–5 years in comparison with the 1950s when it was estimated to take 25 years for such an expansion [68]. Enhancement in dental knowledge revealed genetic, microbiological and immunological mechanisms at the base of periodontal diseases. Point-of-care (POC) testing allows rapid diagnostic tests in which results can be obtained immediately rather than waiting days for outside lab results to arrive [69]. Chairside tests (CSTs) belong to POC cluster of analysis. They can give an immediate indication on the dental health of a single patient to dental operators. CSTs based on saliva, gingival crevicular fluid, cell and bacteria sampling are going to be routinely used by periodontists for a novel approach to the diagnosis, monitoring, prognosis and management of periodontal patients. In the larger healthcare community, ‘dentists and oral health professionals may be positioned to expand the reach and impact of preventive medicine through the application of cost-effective and non-invasive oral fluid screening tests and referring patients for necessary medical care’ [70].

The first cause of tooth loss in industrialised world is periodontitis which is the result of the interaction between genetic tendency and environment influence. In order to understand the growing value of *the 5Ps*, we have to consider some data that are currently arising:

1. The European population is becoming progressively older.

2. Periodontitis generally strikes people older than 40 years.

3. Periodontitis can cause serious detriment of the stomatognathic organ.

It appears clear, therefore, that periodontitis has to be considered as a social disease since it affects millions of people in Europe, and consequently, strategies have to be organised by national and international health organisations in order to intercept and treat the disease before it can create serious damages to a large part of the European population. A similar situation has been recorded in the USA in which 31% of the population exhibited mild forms of periodontitis, 13% displayed periodontitis of moderate severity and 4% suffered from advanced periodontitis [71]. In order to face this situation, we should modify our approach towards diseases. Today, the work of periodontists is considered as ‘a reactive effort’ in the sense that we wait until the patient is sick before responding; on the contrary, the futuristic 5Ps focuses on the early integrated diagnosis (genetic, microbiology, host-derived biomarker detection) with the intention to detect periodontitis at an earlier stage, when it is easier to be treated successfully.

**Figure 30:**
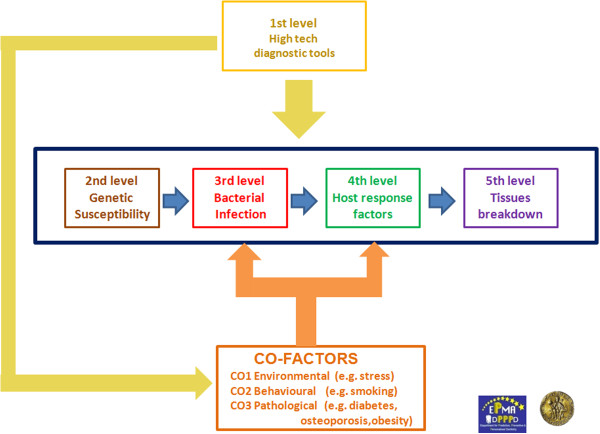
**5Ps flow chart.** Five levels characterise a futuristic approach for periodontal diagnosis. The first level is represented by high-tech diagnostic tools such as LOC and CBCT. In the next future, LOC will be able to give us genetic, microbiological and host-derived information in real time. Co-factors (e.g. diabetes, osteoporosis) will be detected by the use of dedicated high-tech chairside diagnostic tools. Moreover, a detailed bone tissue morphology is revealed by low-radiation digital computed tomography which offers a digital volume composed of three-dimensional voxels that can then be manipulated with specialised software. The second level will provide useful information about the genetic susceptibility of a single patient, while the third level will give us the presence of causative bacterial factors in dental plaque. Finally, host-derived biomarkers (host response factors and factors derived from periodontal tissue breakdown) will be chairside-detected in order to early intercept periodontal destruction.

Here, we intend to propose five diagnostic levels (high-tech diagnostic tools, genetic susceptibility, bacterial infection, host response factors and tissue breakdown-derived products) to be evaluated with the intention to obtain a clear picture of the vulnerability of a single individual to periodontitis in order to organise patient stratification in different categories of risk (Figure 30).

##### First diagnostic level: (lab-on-a-chip, gas chromatographs, cone beam computed tomography)

High-tech diagnostic tools will give periodontists the possibility:

1. To identify a periodontal initial lesion when it is not yet clinically detectable.

2. To intercept the so called ‘active phase’ of periodontitis.

Lab-on-a-chip prototypes, gas chromatographs and cone beam computed tomography are three categories of high-tech devices that will be used everyday for the diagnosis of periodontitis in the not too distant future.

##### Lab-on-a-chip

**Figure 31:**
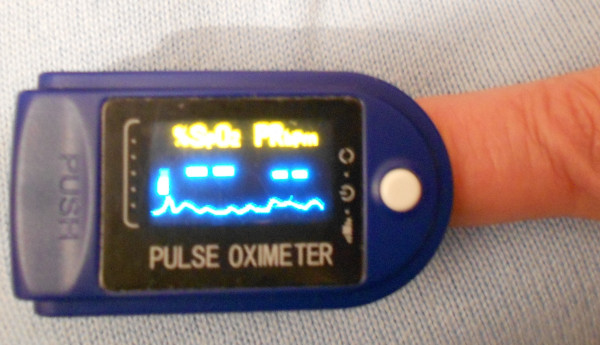
**Lab-on-a-chip micronised pulse oximeter.** Until a few years ago, the diagnostic tool shown in the picture was sensibly bigger than the current one, and for this reason, it could be used only in hospitals. Nowadays, thanks to the reduced dimensions, the oximeter can be lent from hospitals to patients, who can so check daily their oxygen absorption in their own houses.

**Figure 32:**
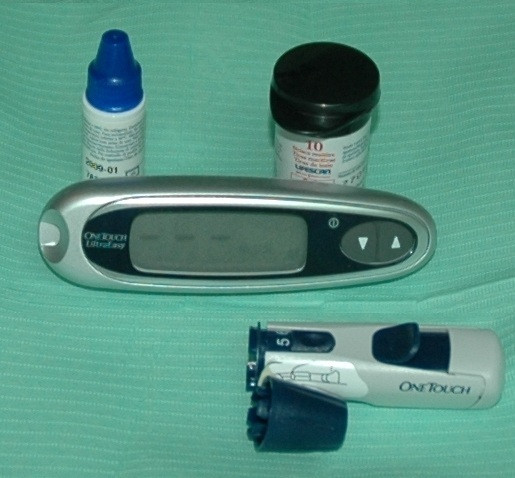
**Probably the first chairside lab-on-a-chip utilised was the illustrated tool to check glycaemic level.** This instrument can be useful for initial diabetes screening in patients at risk. By the use of the patient's single blood drop, the operator can inspect, in a few minutes, the actual glycaemic level in the patient's blood.

A lab-on-a-chip (LOC) is a device that integrates several laboratory functions on a single chip of only millimetres in size (Figures 31 and 32).

LOCs deal with the handling of extremely small fluid volumes down to less than picolitres (microfluidics). Microfluidics represent the technology behind a new miniaturised analysis system for biological applications such as DNA amplification, purification and separation [72]; sequencing [73]; proteomic analysis [74]; and single-cell gene expression profiling [75]. The use of microfluidic devices has a number of significant advantages such as smaller sample requirement (usually several nanolitres), reagents come with the chip and reduced reagent consumption (especially significant for expensive reagents, which is an important concern in clinical laboratories today) that means an immediate indication on the periodontal health of a single patient to dental operators [76]. Finally, the fabrication techniques used to construct microfluidic devices are relatively inexpensive and very open to mass production.

##### Gas chromatographs

Halitosis is a major concern to the general public and the source of a multi-million-dollar industry worldwide [77]. Many patients affected by oral malodour often remain completely unaware of this fact, while others complain of halitosis even if no objective basis can be found: this situation has been defined as the ‘bad breath paradox’. Halitosis is caused by physiologic or pathologic conditions. Physiologic halitosis (the so-called ‘morning breath’) is caused by the stagnation of saliva that disappears with drinking, consumption of food or tooth brushing.

Pathologic halitosis is principally caused by volatile sulphur compounds (VSCs), a family of catabolites resulting from oral bacterial activity. The most important determinants of malodour are hydrogen sulphide (H_2_S) and methyl mercaptan (CH_3_SH), which are catabolites of cysteine and methionine. Other volatile components are aromatic compounds resulting from the degradation of tryptophan (indole and skatole), short-chain fatty acids (acetic and propionic) and some polyamines (cadaverine and putrescine) (Table 4) [78]. The production of volatile sulphureous compounds is mainly derived by the putrefaction of food debris, cells, saliva and blood within the oral cavity mainly through microbial activity [79]. The intensity of clinical bad breath is significantly associated with the amount of intra-oral VSCs [80]. Gram-negative bacteria are the primary pathogens responsible for oral malodour production [81]. Patients with periodontal diseases often complain of oral malodour since the periodontal pocket is an ideal environment for VSC production with respect to the bacterial profile and sulphur source. Other authors demonstrated that a higher amount of VSCs was highly correlated with probing pocket depth, clinical attachment level, bleeding on probing, radiographic bone loss and Gram-negative pathogen species (*T. denticola*, *P. gingivalis*, *P. intermedia*) [82]. The most common device used to evaluate halitosis is the Halimeter® (Interscan Corp., Chatsworth, CA, USA) that measures volatile sulphur compounds in exhaled air. The Halimeter® does not measure other important odorants, such as volatile fatty acids and cadaverine, which are involved in oral halitosis: this could lead to a false negative result when malodour can be detected by the examiner, but the volatile sulphur compound levels are in the low range [83]. A portable gas chromatograph named Oral Chroma™ (Abilit Corp., Osaka, Japan) has been introduced to detect VSCs [84].

**Table 4:** Principal volatile components responsible for oral pathologic halitosis

**Volatile components**	**Compounds**
Volatile sulphur compounds	Hydrogen sulphide, methyl mercaptan
Aromatic compounds	Indole, skatole
Short-chain fatty acids	Acetic, propionic
Polyamines	Cadaverine, putrescine

The Halimeter® has been shown to be more sensitive to H_2_S than to methyl mercaptan and almost insensitive to dimethyl sulphide, whereas the Oral Chroma™ measures all three gases with equally high sensitivities [85].

##### Cone beam computed tomography

**Figure 33:**
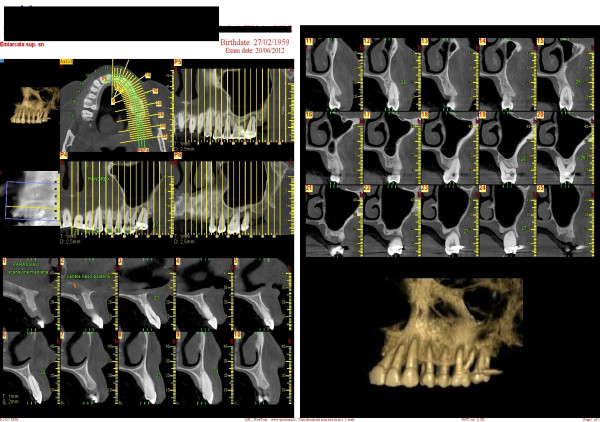
**MSCT.** It collects the anatomical data and produces a digital volume composed of three-dimensional voxels that can then be visualised and manipulated with specialised software. A three-dimensional reconstruction of the upper and lower maxillae can be obtained, and anatomical structures can be easily inspected.

Multi-slice computed tomography (MSCT) is a medical imaging technique using a narrow fan beam that rotates around the patient's head acquiring thin axial slices. During these repeated rotations, MSCT emits a high radiation dose, and it leaves a gap of information between each rotation. Consequently, the software must connect together the images and calculate what is missing. Cone beam computed tomography (CBCT) technology was first introduced in the European market in 1996 and into the US market in 2001 [86]. CBCT uses a cone-shaped beam (the X-rays are divergent) to acquire the entire image in a scan using only one rotation. During a CBCT scan, the scanner rotates around the patient, obtaining up to almost 600 separate images. The scanning software collects the anatomical data and produces a digital volume composed of three-dimensional voxels (instead of traditional pixels) that can then be visualised and manipulated with specialised software. The result is a more precise image without missing information (Figure 33).

##### Second diagnostic level: genetic susceptibility

The largest part of the studies shows no correlations between the presence of disease markers and the tested SNPs in both the aggressive and chronic forms of periodontitis [14]. The polymorphisms that seemed to be linked with periodontitis in different ethnic groups were associated with the Fc-gamma receptor genes. However, these polymorphisms of the same gene were found in both chronic periodontitis and aggressive periodontitis [87,88]. A weak association between the SNP in interleukin-1 genes and chronic periodontitis was found in a recent meta-analysis [89]. Interleukin-1 is a pro-inflammatory agent that is released by macrophages, lymphocytes, platelets and endothelial cells. The gene encoding this cytokine is assigned to chromosome 2q13–21 [90].

In 1997, Kornman et al. described a composite genotype formed by two polymorphic loci - interleukin-1A (−889) and interleukin-1B (+3954) - which are single-nucleotide polymorphisms that carry a C-T transition [91]. Interleukin-1A (−889), however, was outdated by the investigation of the interleukin-1A (+4845) G-T dimorphism, in which the two loci comprising the periodontitis-associated genotype were found to be in linkage disequilibrium [92].

**Figure 34:**
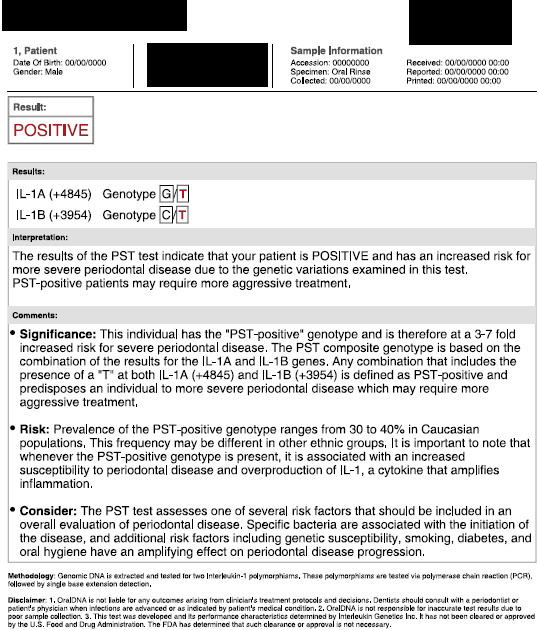
**PST-positive genotype test interleukin-1A (+4845) and interleukin-1B (+3954).** Several studies encourage the routine application of such tests to assess periodontal risk in a single patient.

Therefore, the analysis of the interleukin-1A (+4845) single-nucleotide polymorphism provides the same genetic information [93]. Several studies have evaluated the utility of the commercially available IL-1 genetic susceptibility test (Figure 34) [94]. Unfortunately, we do not have any sufficient size-controlled studies that would allow us to evaluate the efficacy of the IL-1 genotype [95,96]. Thus, although certain studies are encouraging, there are currently insufficient data to support a modification of treatment protocols for chronic periodontitis patients based on IL-1 testing [97].

##### Third diagnostic level: bacterial infection

Haffajee and Socransky [98] suggested six types of lines of evidence to be used to support an aetiological role for bacteria in periodontal infections:

1. Elevated odds ratio in disease.

2. Conversion of disease to health when bacteria are suppressed.

3. Development of a host response.

4. Presence of virulence factors (capability to avoid host defences and to damage tissues).

5. Evidence from animal studies corroborating the observations in humans.

6. Support from risk assessment studies.

Following the above criteria, the consensus report of the World Workshop on Periodontitis [99] identified three bacterial species for which sufficient data have accumulated as *causative factors* for periodontitis: *A. actinomycetemcomitans* (recently renamed to *Aggregatibacter actinomycetemcomitans*) [100], *P. gingivalis* and *Bacteroides forsythus* (renamed to *Tanerella forsythia*) [101]. The consensus report stated that *A. actinomycetemcomitans* is most often found in aggressive (‘early-onset’) periodontitis, whereas *P. gingivalis* and *T. forsythia* are found more frequently in chronic (‘adult-onset’) periodontitis. *Moderate evidence* to support an aetiological role was reported for *C. rectus*, *E. nodatum*, *P. intermedia*, *P. nigrescens*, *Parvimonas micra* (formerly *Micromonas micros* and *Peptostreptococcus micros*), the *Streptococcus intermedius* complex and *T. denticola*. Finally, an *initial evidence* included on the list of probable periodontal pathogens *E. corrodens*, enteric rods, *Pseudomonas* species, *Selenomonas* species and *Staphylococcus* species. This report received general acceptance by the periodontal community and is still regarded as valid.

Even if there are no sufficient microbiological evidences that could help us in distinguishing the different forms of periodontitis, it is clear that:

•The chronic and aggressive forms of periodontitis are not monoinfections.

•Some microbiota are more important than others as etiological agents of periodontitis.

For these reasons, it appears clear that the microbial testing of sub-gingival plaque could be a valid support for a correct diagnosis of periodontitis. The anaerobic culture test is the most sophisticated technique to analyse the composition of sub-gingival plaque. All cultivable microbial species in the sub-gingival sample can be detected, and proportions of the various pathogens can be established. Anaerobic culture testing allows the antimicrobial susceptibility testing of periodontal pathogens. Anaerobic culture testing is advised especially in the case of refractory periodontitis, atypical forms of pathogens or periodontitis, peri-implantitis and immunocompromised patients. In routine cases, a DNA-based chairside test (semi-quantitative polymerase chain reaction (PCR)) is indicated. Bacteria do not need to be viable; consequently, time is not an issue with the present test. The number of target bacteria is determined semi-quantitatively (0 to +++).

CSTs for bacteria detection provide information about the presence and relative importance of putative pathogens.

The periodontist has to follow the following steps in order to perform a correct DNA-based chairside test (semi-quantitative PCR) for bacterial plaque analysis:

•Meticulous removal of supra-gingival plaque.

•Sampling of sub-gingival plaque by the insertion of sterile paper points into the deepest pockets in each quadrant.

•Sending samples to a specialised laboratory.

**Figure 35:**
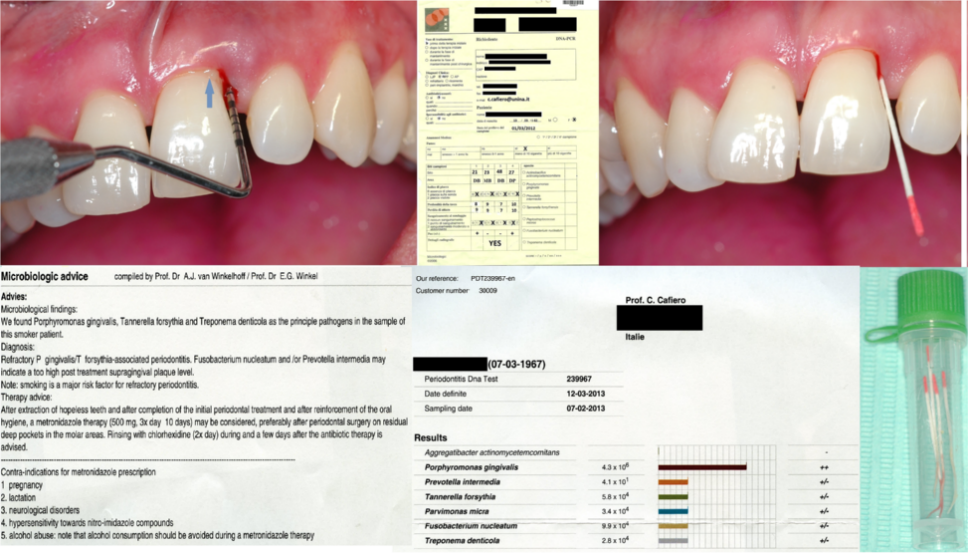
**Consecutive steps to perform a correct DNA-based chairside test (semi-quantitative PCR) for bacterial plaque analysis.** Clinical diagnosis (chronic periodontitis or aggressive periodontitis), cigarette smoking, systemic pathologies and antibiotic consumption are the initial information requested. Then, a meticulous removal of supra-gingival plaque has to be performed. After that (see the figure clockwise), periodontal charting detects four different sites showing the deepest probing pocket depth whose values were reported on a DNA-PCR form. Clinical attachment level measures of the selected sites are also requested. In the present case, pyorrhea was present (*blue arrow*). Samples of sub-gingival plaque are carried out by the insertion of sterile paper points into the deepest pockets in each quadrant. Paper points are then stored into a vial and the samples sent to a specialised laboratory to perform the DNA examination and identification of bacterial species, together with the DNA-PCR form. The number of target bacteria is determined semi-quantitatively (0 to +++) and sent to the periodontist together with the diagnosis and therapy advice. Results are useful for the periodontist who will have a picture of a single patient's microbiological infection and for the patients in order to reinforce their compliance. Finally, the periodontist, having considered the species found, should propose an individually tailored maintenance care programme to a single patient.

The specialised laboratory will perform the DNA examination and identification of bacterial species. The number of target bacteria is determined semi-quantitatively (0 to +++) (Figure 35). Since optimal plaque control by the patient is of paramount importance for a favourable clinical and microbiologic response to therapy, microbiological analysis laboratory results should be discussed with patients in order to reinforce their compliance. Patients have to be placed on an individually tailored maintenance care programme, including the instruction of oral hygiene, in order to obtain optimal plaque control and continuous evaluation of the occurrence of disease progression.

##### Fourth and fifth diagnostic levels: host response factors and tissue breakdown-derived products

At present, well-studied molecules associated with host response factors and with derived tissue destruction mediators have been proposed as diagnostic biomarkers for periodontitis [102]. Many dental associations, such as the American Dental Association (ADA), recognise the importance of continued research on oral fluid diagnostics and welcome the development of rapid point-of-care tests that provide accurate measurements of clinically validated biomarkers. The ADA council ‘encourages dentists to take leadership roles in integrating the tests and related technologies into clinical practice, consistent with the best available scientific evidence’ [70].

We are going to discuss inflammatory/immunological reaction and sub-clinical tissue destruction in the same section since they:

1. Happen approximately at the same time.

2. Share the same modality of non-invasive sample collection.

3. Release biomarkers which can be detected in the same diagnostic medium (oral fluid, gingival crevicular fluid)

##### Oral fluid (whole saliva) as a diagnostic tool

Probably the simplest organic diagnostic tool is oral fluid, a watery substance with multiple functions. Oral fluid or whole saliva is composed of 99.5% water, while the other 0.5% consists of antibacterial compounds. The advantages of using oral fluid as a diagnostic medium for a rapid point-of-care testing include non-invasive sample collection, simplicity of access and acceptance by patients. Oral fluid (also called as *whole saliva*) is the fluid obtained from the mouth by expectoration. It includes glandular-duct saliva and gingival crevicular fluid:

1. Glandular-duct saliva: saliva secreted by the parotid, sub-mandibular, sub-lingual and minor salivary glands (2,000 ml/24 h) is obtained directly from the glandular ducts with specially designed collectors. Glandular-duct saliva contains predominantly secretory IgA.

2. Gingival crevicular fluid (GCF) is an exudate flushing from the gingival sulcus (0.5 to 2.5 ml/24 h). GCF is a versatile and non-invasive means to sample the biomarkers of inflammation and bone resorption in the oral cavity. GCF represents serum components overlaid with products from local physiologic or pathologic phenomena. In particular, pathologic phenomena such as connective tissue destruction and bone loss may have a diagnostic value [103,104]. Whilst gingival crevicular fluid is the most appropriate diagnostic medium to use in analyses, it appears clear that the use of whole saliva is more practical even if reactants need to be highly sensitive since biomarkers are more diluted [105,106].

### Salivary biomarkers for periodontal disease

Recently, the entire human salivary proteome was reported by a consortium of three research groups [107], and this revealed that 1,166 proteins are present in human saliva [108]. Over 65 GCF components have been examined as possible markers for the progression of periodontitis (for a complete review, see [109]). These components fall into three general categories: (1) host-derived enzymes and their inhibitors, (2) inflammatory mediators and host response modifiers and (3) tissue breakdown products. We have searched the literature for more promising components of gingival crevicular fluid in regard to potential diagnostic value for periodontitis (Table 5) (for a complete review, see [110]).

**Table 5:** Most promising salivary biomarkers for the diagnosis of periodontal disease

**Salivary biomarkers**	**Components**
Host-derived enzymes	Alkaline phosphatase
	Beta-glucuronidase
	Cathepsin B
	MMP-8 (collagenase-2)
	MMP-9 (gelatinase)
	Dipeptidyl peptidases II and IV
	Elastase
Host response modifiers	RANKL
	OPG
	RANK
Tissue breakdown products	1-CTP
	C-4-S

#### Alkaline phosphatase (host-derived enzyme)

Alkaline phosphatase is an enzyme produced principally by neutrophils and then by fibroblasts, osteoblasts, osteoclasts and several bacteria. It plays a role in the physiological turnover of the periodontal ligament, root cement and alveolar bone. The amount of alkaline phosphatase in gingival crevicular fluid samples appears higher in the active sites than in the inactive sites. Moreover, elevated alkaline phosphatase levels preceded attachment loss, while no clinical parameters were yet discriminatory [111].

#### Beta-glucuronidase (host-derived enzyme)

Beta-glucuronidase is a lysosomal enzyme that could be thought as an indicator of periodontal disease activity. Lamster et al. [112] showed a predictive value for beta-glucuronidase in relation to clinical attachment loss. Nakashima et al. [111] reported that beta-glucuronidase was significantly higher in active vs. inactive sites.

#### Cathepsin B (host-derived enzyme)

Cathepsin B is an enzyme active in proteolysis. Macrophages are the cellular source of cathepsin B in gingival crevicular fluid [113]. Cathepsin B levels (1) have been found to be increased in periodontitis but not in gingivitis, (2) were higher in rapid loss sites than in paired control sites and (3) appeared reduced after periodontal treatment [114-116].

#### MMP-8 (collagenase-2) (host-derived enzyme)

MMP-8 in gingival crevicular fluid has latent and active forms. The latent enzyme may be present in gingivitis and the active form in periodontitis. MMP-8 appears 18-fold higher in progressing periodontitis vs. stable periodontitis [117]. Mancini et al. proposed the use of MMP-8 levels in gingival crevicular fluid as a test for active periodontal destruction [118].

#### MMP-9 (gelatinase) (host-derived enzyme)

MMP-9 appears elevated in subjects affected by advanced periodontitis associated with red complex anaerobic periodontal pathogens (e.g. *P. gingivalis* and *T. denticola*) [119]. Samples from patients with recurrent attachment loss showed a twofold increase of mean active MMP-9, and these levels decreased significantly following adjunctive metronidazole therapy [120].

#### Dipeptidyl peptidases II and IV (host-derived enzyme)

Neutrophils, lymphocytes, macrophages and fibroblasts are the main sources of dipeptidyl peptidases II and IV. Their main function lies in the activation of the pro-forms of cytokines and enzymes and in the degradation of collagen tissue. Higher levels of both enzymes in sites with rapid and gradual attachment loss were reported with respect to sites without attachment loss [121].

#### Elastase (host-derived enzyme)

Elastase is a proteinase released from the azurophilic granules of neutrophils and from macrophages (also called MMP-12). Elastase has been recorded in GCF from periodontal patients at elevated levels and reduced after periodontal treatment. Many authors [122-124] observed higher elastase levels in sites demonstrating progressive attachment loss in comparison with inactive sites.

#### RANKL/OPG/RANK system (host response modifiers)

The RANKL/OPG/RANK system can be detected in the gingival tissue, GCF and saliva. In the course of periodontitis, RANKL is secreted by osteoblasts, fibroblasts, bone marrow stromal cells and activated T and B cells. Under physiological condition, RANKL produced by osteoblasts binds to RANK on the surface of pre-osteoclasts. RANKL is up-regulated by osteotropic factors such as OPG. RANKL is increased whereas OPG is decreased in periodontitis compared to healthy gingiva or gingivitis [125].

#### 1-CTP (tissue breakdown products)

Pyridinoline cross-links represent a class of collagen-degrading molecules that include pyridinoline, deoxypyridinoline, N-telopeptides and C-telopeptides. The role of pyridinoline cross-linked carboxyterminal telopeptide of type I collagen (1-CTP) levels in gingival crevicular fluid as a diagnostic marker of periodontal disease activity has been investigated by several studies. High levels of 1-CTP were strongly correlated with clinical parameters and putative periodontal pathogens. Results showed that 1-CTP appeared as a good predictor of future alveolar bone and attachment loss and demonstrated significant reductions after periodontal therapy [126].

#### C-4-S (tissue breakdown products)

Chondroitin-4-sulphate (C-4-S) is the most common glycosaminoglycan in untreated chronic periodontitis sites, as shown in both animal and human studies. Elevated glycosaminoglycan concentrations were also found in aggressive periodontal diseases, and associations have been made with periodontal pathogens such as *P. gingivalis*[126]. A statistically significant correlation between the GCF content of C-4-S, a bone-specific glycosaminoglycan, and PPD and CAL was reported [127].

## Conclusions

Oral fluid is the mirror of periodontal health. It is a medium for clinically relevant information since it contains biomarkers specific for periodontal diseases. Although the periodontal diagnostic value of oral fluid has been recognised for some time, most scientific papers in the recent past have failed to support consistent aids to the clinician in periodontal diagnosis and therapy. Advances in microfluidics technology are revolutionising molecular biology procedures for enzymatic analysis, DNA analysis and proteomics. The evolution of microfluidics, *digital microfluidics*, appears promising for future application to diagnose periodontal diseases and to prognosticate periodontal treatment.

**Figure 36:**
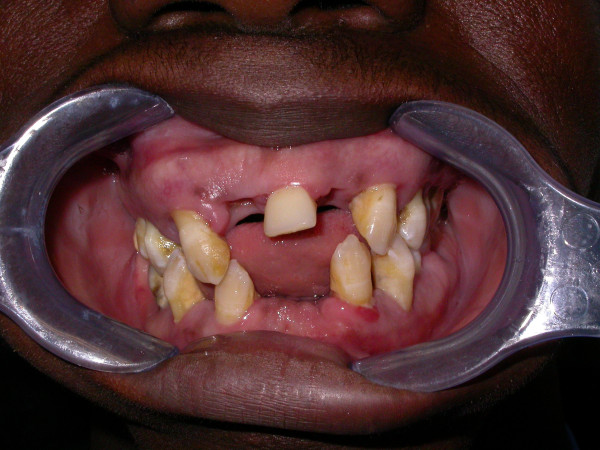
**A 24-year-old patient from Nigeria suffering from generalised aggressive periodontitis.** Periodontal diagnosis was effected in Naples (Italy) when the disease had already destroyed up to 80% of the periodontal supporting bone.

**Figure 37:**
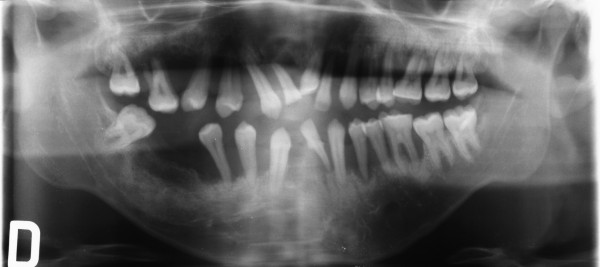
**A panoramic X-ray of the same patient.** An advanced generalised destruction of the supporting bone tissue is evident. One of the most important topics in periodontal diagnosis in the next future will be to create microfluidic chips allowing healthcare providers in poorly equipped hospitals and areas of the world.

Lab-on-a-chip technology may soon become an important part of efforts to improve worldwide periodontal health [128]. In developed nations, the most highly valued qualities for portable, easy-to-use diagnostic tools include speed, sensitivity and specificity; while in the underserved communities, resource-poor areas and poor countries, the goal of researchers is to create microfluidic chips that will allow healthcare providers in poorly equipped hospitals [129] (Figures 36 and 37).

The use of LOC devices for periodontal inspection will involve less education than current diagnostic procedures and allow patients to be screened for periodontal disease in settings other than the periodontist practice, such as at general practitioners, general dentists or dental hygienists [130].

All these benefits make the lab-on-a-chip technology ideal for predictive, preventive, personalised and participatory periodontology. The 5Ps represents with no doubt the future of our profession. Personalised therapy with tailored respect to the particular medical reality of the specific stratified patient will be the ultimate target to be realized by the 5Ps approach. A long distance has to be covered to reach the above targets, but the pathway has already been clearly outlined: it is ‘time for new guidelines in advanced healthcare’ in dentistry too [131].

## Consent

Written informed consent was obtained from the patients for the publication of this report and any accompanying images.

## Competing interests

The authors declare that they have no competing interests.

## Authors’ contributions

CC and SM conceived the present paper and participated in its draft. Both authors read and approved the final manuscript.

## Authors’ information

CC is a researcher and professor at the University of Naples “FEDERICO II”. SM is a full professor and the Chairman of the Degree Course in Dentistry at the University of Naples “FEDERICO II”.
